# Current Concepts of Epigenetics and Its Role in Periodontitis

**DOI:** 10.1007/s40496-017-0156-9

**Published:** 2017-11-06

**Authors:** Lena Larsson

**Affiliations:** Department of Periodontology, Institute of Odontology, Sahlgrenska Academy, University of Gothenburg, Box 450, SE-405 30 Gothenburg, Sweden

**Keywords:** Epigenetics, Periodontitis, DNA methylation, Histone

## Abstract

**Purpose:**

The focus of this review is to provide an overview of the recent findings on the role of epigenetic mechanisms in periodontal disease, including disease susceptibility, progression, and as potential treatment options.

**Recent Findings:**

The findings on the influence of oral pathogens on epigenetic regulation of pathogen recognition receptors, such as Toll-like receptors, as well as pro-inflammatory cytokines suggest an important role for epigenetics in the regulation of the host immune response. Recent studies also show that the epigenetic pattern in periodontitis lesions differ from that of healthy and gingivitis tissue. In addition, these patterns differ between tissues in the same individual. Research is also indicating a role for both DNA methylation and histone acetylation on cells osteogenic differentiation and bone regeneration.

**Summary:**

Knowledge of epigenetic pattern in periodontal diseases may add not only to the knowledge of susceptibility of the disease but may also be a diagnostic tool to identify patients at risk to develop the severe form of periodontitis. In addition, recent research within gene therapy and tissue engineering indicate a role for epigenetics also to improve regeneration of periodontal tissues.

## Introduction

Periodontitis is a disease characterized by chronic inflammation in the gingival tissues in response to bacteria colonizing the tooth surface and gingival crevice. In certain individuals, this immune response results in tissue destruction and loss of alveolar bone and connective tissue [1]. Periodontitis is one of the most common diseases in humans, and the severe forms affect about 10% of the adult population [2, 3]. The severe form of periodontitis was recently shown to be the sixth-most prevalent condition worldwide [4]. Genetic polymorphisms, environmental risk factors (bacteria), lifestyle factors (smoking, diet), and epigenetic factors have been suggested to contribute to the severity of the disease and the susceptibility of an individual to develop periodontitis [5, 6]. Epigenetics consider the interface between genetics and environmental/lifestyle factors. An alteration in the epigenetic pattern may contribute to the individual differences in local gene expression associated with inflammation and susceptibility for disease [7]. Epigenetics in the field of dental research is still at an early stage, but reports related to inflammation and inflammatory markers have emerged, as well as reports on the influence of environmental factors affecting oral health [8, 9]. The focus of this review is to provide an overview of the recent findings on the role of epigenetic mechanisms in periodontal disease, including disease susceptibility, progression, and as potential treatment models.

## Epigenetics

The term epigenetics was coined by Conrad H. Waddington already in the 1940s [10] as a term to describe the “causal mechanisms” by which genes give rise to the phenotype. The modern definition of epigenetics is changes in gene expression that are not encoded in the DNA sequence, including chemical alterations of DNA and its associated proteins, leading to remodeling of the chromatin and activation or inactivation of a gene [11]. In contrast to our genome that is the same in all cells and throughout our life, our epigenome is dynamic and differ between cells and tissues. In addition, it changes during life in response to changes in the cellular microenvironment as well as to environmental factors.

**Fig. 1:**
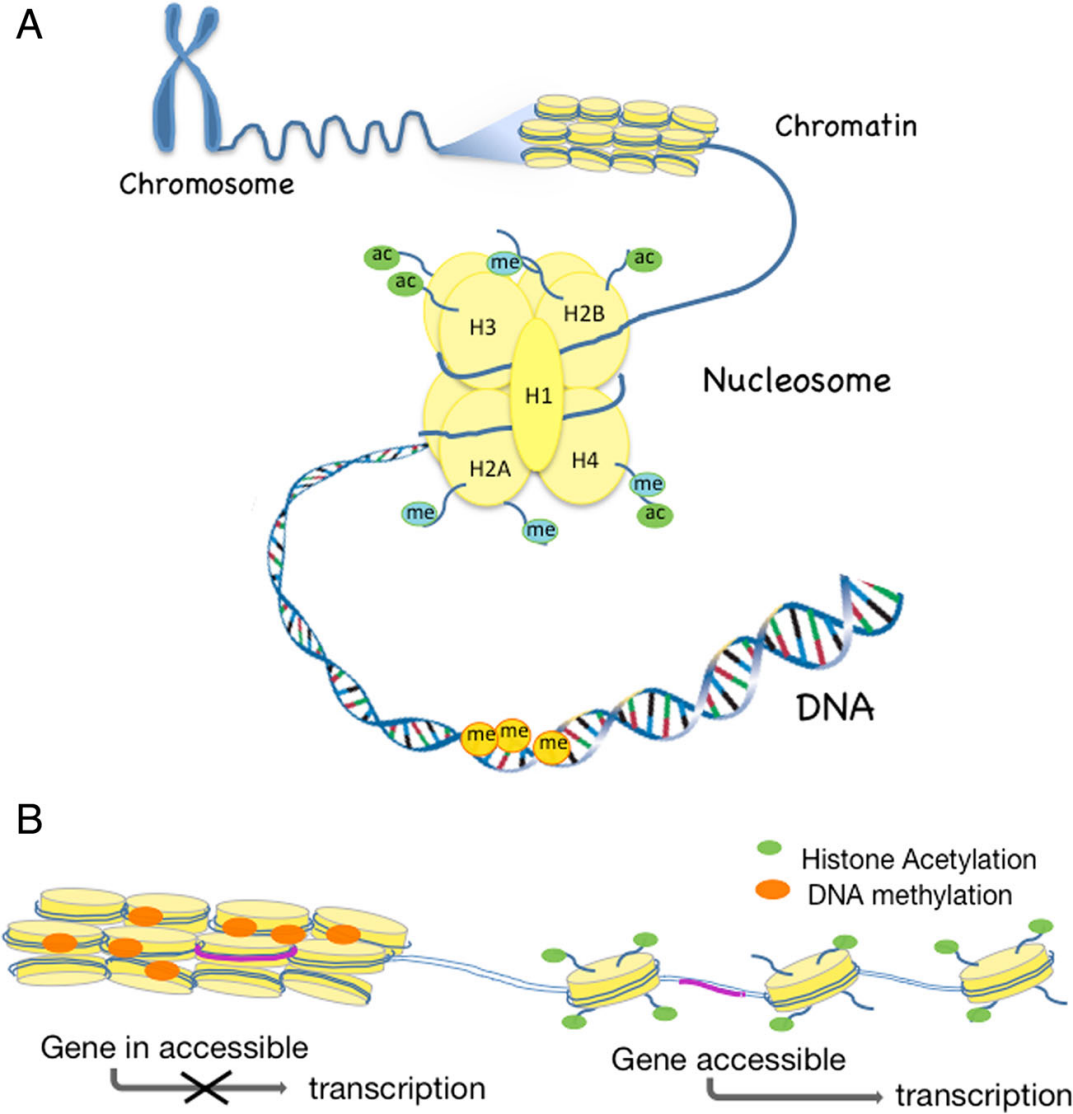
Schematic overview of the structure of the chromatin (**a**) and the epigenetic modifications of DNA methylation and histone modifications and their influence on chromatin formation and gene expression (**b**). Ac acetylation, Me methylation

Our genetic material in form of the DNA helix is packaged in the nucleus as chromatin. Epigenetic mechanisms play a vital role in the regulation of the structure of the chromatin. The chromatin can be loosely packed and available for gene expression or as very densely packed chromatin leading to silencing of gene expression [12]. The building blocks of chromatin are the nucleosomes, which consists of 146 bp of DNA and a histone complex. The histone complex in turn, includes two copies each of histones H2A, H2B, H3, and H4 and a linker histone H1 that connects the nucleosomes forming the primary chromatin structure (Fig. 1). Histones can be acetylated or methylated at amino acid tails that protrude from the nucleosome [12]. DNA methylation refers to the addition of methyl groups to cytosine bases (5mC) at specific sites in the DNA sequence (i.e., CpG sites), by DNA methyltransferases (DNMTs) [11, 13]. Furthermore, to add another level to the concept of DNA methylation, 5mC can be oxidized into 5-hydroxymethylcytosine (5hmC) by the ten-eleven translocation (TET) family of enzymes [14]. The biological function of 5hmC is not clear but has been suggested to be a mechanism for demethylation of DNA and re-activation of genes [15]. Both DNA methylation and modifications of the histones alter the configuration of the chromatin resulting in changes in gene expression. Transcriptionally active genes are associated with low levels of DNA methylation and high levels of histone acetylation. Histone modifications and DNA methylation are not separate events but are linked resulting in a unique tissue and cell-specific gene expression (Fig. 1) [7].

## Recent Findings on The Role of Epigenetics in Periodontitis

Chronic inflammatory diseases, such as periodontitis, have specific target tissue in which the inflammation is persistent and tissue destruction occurs. A periodontitis patient may have only some teeth affected by disease indicating a different response to bacteria and/or a difference in microbiomes at different sites in the oral mucosa hence, suggesting a local change in the regulation of genes associated with inflammation. It has been suggested that epigenetic changes occur locally at the biofilm-gingival interface around the teeth and that epigenetic modifications differ between an inflamed periodontitis site and non-inflamed sites in the same individual [7, 16].

An infection and the host immune response may induce changes in the epigenome that in turn may add to the susceptibility to disease. The oral epithelial cells are the first line defense towards pathogens, and it may be hypothesized that the presence of bacteria induce alterations of the epigenome in these cells that subsequently also affects inflammatory cells, by inducing changes in the signaling pathways and gene expression. Indeed, oral pathogens, such as *Porphymonas gingivalis* (*P. gingivalis*) and *Fusobacterium nucleatum* (*F. nucleatum*), are able to induce acetylation of histones and down-regulation of DNMT1. In addition, activation of pathogen recognition receptors (PRRs) and Toll-like receptors (TLRs) by these bacteria further induced histone modifications in oral epithelial cells [17•].

TLRs play an important part in the innate immunity through their ability to recognize so called pathogen-associated molecular patterns (PAMPs), including bacterial lipopolysaccharide (LPS). Dysregulation of TLR expression may influence the host response against periodontal pathogens leading to an increase in inflammation and susceptibility to periodontitis [18]. The DNA methylation pattern of *TLR2* and *TLR4* has previously been investigated in gingival biopsies [19, 20]. The *TLR4* was reported to be unmethylated in both healthy subjects and periodontitis patients. In contrast, the methylation pattern for *TLR2* was not so clear containing both methylated and unmethylated parts of DNA [19]. However, de Faria Amormino and co-workers [20] found a higher degree of methylation in *TLR2* in samples from periodontitis patients compared to controls. Interestingly, a correlation between *TLR2* methylation and the number of inflammatory cells within the connective tissue was reported. This indicates that differences in DNA methylation level between studies may be a result of the level of inflammation in the tissue samples included. In a recent study, the DNA methylation pattern of *TLR2* was investigated in human gingival epithelial cells (HGECs). It was shown that growing epithelial cells in the presence of *P. gingivalis* induced DNA methylation. A similar finding was also found in the gingiva of mice treated with *P. gingivalis* [18]. An activation of TLRs by periodontal pathogen not only induced histone acetylation in oral epithelial cells but also activation of transcription factor Nuclear factor-κb (NFκB) [17•]. NFκB is a transcription factor that activate and co-ordinates the innate immunity as well as participate in osteoclast differentiation and induction of Matrix metalloproteinases (MMPs) and adhesion molecules [17•, 21]. Interestingly, DNA methylation CpG sites around NFκB binding site in the *TLR2* promoter region have been identified. It may be speculated that alterations in DNA methylation can affect the binding of NFκB to the promoter hence, influencing activation and regulation of TLR expression [19].

Long-term treatment of human periodontal fibroblast (PDL) cells with *P. gingivalis* was found to induce hypermethylation of several genes related to extracellular matrix (ECM). It was hypothesized that the decrease in the expression of these ECM-related proteins due to hypermethylation reduce the osteoblastic differentiation and decreased expression of bone-specific proteins in PDL cells. In addition, the decrease in expression of cell surface receptors influenced adhesion of cells to the ECM, which further lead to an initiation of expression of MMPs [22]. MMPs are key factors in matrix degradation and bone resorption as well as in wound healing, cell proliferation, inflammation, and immunity [23, 24]. In line with these findings, *Treponema denticola* (*T. denticola*) was found to induce hypomethylation of the *MMP2* promoter and a chronic activation of pro-MMP2 in PDL cells [23]. This indicates that *T. denticola* through epigenetic mechanisms play a role in activation and enhancement of the loss of supporting tissue seen in periodontitis. In summary, these studies add to the knowledge on how dysbiosis epigenetically influence molecular signaling within the host immune response as well as on how the local microenvironment around cells can play a part in regulating activation and/or production of signaling molecules that further influence tissue degradation and uphold a chronic inflammation. Interestingly, variations in the DNA methylation pattern between healthy individuals and periodontitis patients have been reported to be higher in genes related to immune response [25].

The DNA methylation levels of CpG sites in 22 inflammatory genes were analyzed in gingival tissue samples from patients with aggressive periodontitis and controls. A decrease in methylation level was found in promoter regions of *IL17C* and *CCL25* in periodontitis patients indicating an increase in gene expression [26]. The levels of difference in DNA methylation found in this study were similar to those reported by Barros and Offenbacher [16, 26]. The IL17C and CCL25 cytokines play an important part in the immune response to bacteria and in the TH17 cell immune response. It was suggested that changes in their methylation pattern and subsequent increase in gene expression might add to the loss of periodontal attachment in periodontitis [26].

Several inflammatory cytokines and markers have been investigated regarding their epigenetic pattern and gene regulation [7]. The *IL-6* promoter was found to be partially methylated in gingival tissue samples from both periodontitis patients and healthy individuals, but the expression of IL-6 was higher in periodontitis patients [27]. In a recent report, no difference in methylation level of the *IL-6* promoter was found between periodontitis patients and healthy controls [28]. Analysis of 12 CpG sites in the *TNFα* promoter in patients with chronic periodontitis and healthy controls revealed differences in DNA methylation only at one CpG site [29]. A comparison of the DNA methylation pattern of inflammatory regulators *SOCS1* and *LINE-1* in patients with aggressive periodontitis with healthy individuals showed a higher degree of hypomethylation in oral epithelial cells in healthy subjects [30]. Similar results were reported by Andia and co-workers [31]. In this study, gingival tissue samples with absence of inflammation obtained from periodontitis patients and healthy controls were included. Interestingly, a previous history of periodontitis did not influence the methylation level of *SOCS1*, *SOCS3*, and *LINE-1*. In addition, intragenic CpG islands in the *SOCS1* gene was hypermethylated in periodontal tissue compared to healthy tissue. However, no difference in gene expression of SOCS1 was found [32••]. Based on the current research, it may be suggested that oral epithelial cells have a different epigenome when present in an inflammatory environment compared to the cells in a non-inflammatory environment. However, the results shown by Planello et al. [32••] suggested that the increase in DNA methylation in the *SOCS1* gene in periodontitis was not due to the presence of inflammatory cells.

In addition to the effect on the expression of inflammatory cytokines and signaling molecules, oral pathogen can also influence bone regeneration. Cells from the periodontal ligament have the potential to differentiate into osteoblasts, and RUNX2 is a key factor in this process. *P. gingivalis* LPS induced an increase of DNMT1 and a down-regulation of RUNX2 expression in human periodontal ligament (HPDL) cells. This indicates that the inhibitory effect of LPS seen on osteoblastic differentiation may be a consequence of DNA hypomethylation of *RUNX2* [33]. Treatment of human gingival fibroblasts with a DNA methylation inhibitor-induced hypomethylation of *RUNX2* and alkaline phosphatase (*ALP*), and a subsequent treatment of these cells with BMP2 induced the expression of RUNX2 and ALP as well as induced differentiation into bone-forming osteoblasts [34•].

The focus of epigenetic studies within periodontal diseases has mostly been on the epigenetic pattern in genes related to inflammation or bone formation. However, there are some studies on the expression of epigenetic markers in periodontal diseases. Martins and co-workers [17•] reported on a down-regulation of DNMT1 and an up-regulation of acetylated histone 3 in the epithelial cells close to the inflammatory lesion in a mice periodontitis model. A significant up-regulation of DNMT1 and TET1 mRNA was found in periodontitis patients compared to healthy controls [25]. The proportion of TET2 positive cells was larger in periodontitis lesions compared to gingivitis lesions [35]. Interestingly, 5mC and 5hmC levels were similar in both groups. The TET2 enzyme is involved not only in the conversion of 5mC to 5hmC but also in the downstream process of demethylation [14]. Therefore, this increase in TET2 together with unchanged 5hmC levels may indicate a higher demethylation activity in periodontitis lesions. Furthermore, suggesting a difference in the epigenetic regulation of gene expression of inflammatory genes and thereby a potential influence of disease severity [35]. Recently, it was found that the hypermethylated CpG in periodontitis were found in enhancer regions and that this hypermethylation did indeed prevent the enhancer activity [32••].

Studies analyzing epigenetic mechanisms in different tissues have shown that these patterns differ even when the tissues come from the same patient. A comparison between gingival tissues and peripheral blood samples from the same periodontitis patient showed that the methylation level of the *IL-6* promoter was lower in gingival tissues. However, the mRNA level of IL-6 was similar in both tissue and blood [28]. The level of global 5hmC DNA methylation level was higher in peripheral blood samples compared to the level in tissue samples from periodontitis patients [35]. In contrast, a larger overall DNA methylation was found in buccal epithelial cells compared to blood cells [36]. These findings are in line with the knowledge that the epigenetic pattern varies between tissues and that it is therefore vital that when investigating these mechanisms one has to consider the best suitable tissue available that corresponds with the disease investigated [26, 36, 37]. For studies on epigenetics and periodontitis, gingival tissues have been suggested as the preferred tissue [26]. It should be noted that gingival tissue samples contain epithelial cells, inflammatory cells, and connective tissue cells, all contributing to the results. Laser capture microdissection presents a method making it possible for analysis of epigenetic pattern of specific cell groups in an inflammatory lesion and has been used for epigenetic analysis of periodontitis tissue [16, 31].

## Clinical Application of Epigenetics Within The Field of Periodontitis

### Diagnostics and Epidrugs

Knowledge of epigenetics contributes to a better understanding of the interactions between genes and the environment and may provide explanations to why patients with the same clinical phenotype respond differently to treatment [8]. To be able to correlate an epigenetic pattern/marker with a clinical phenotype is of interest as well as using epigenetics as a tool to identify patients at risk to develop periodontitis. The possible use of buccal swabs, scraping of the oral mucosa or saliva for epigenetic analysis, makes it clinically feasible as a diagnostic tool. Research on using such samples for epigenetic analysis has so far mostly been done in the field of oral cancer. Oral rinse samples from patients with oral squamous cell carcinoma (OSCC) have been analyzed for chromatin modifications and DNA methylation [38, 39]. DNA methylation has also been investigated in buccal swabs and saliva [40, 41]. At present, only a few studies combining epigenetic analysis with clinical measurements are available. A negative correlation was found between methylation levels of *IL-6* in gingival tissue and probing depth in periodontitis patients [28], compared to the positive correlation with *TLR2* methylation and probing depth [20].

The fact that epigenetic mechanisms are reversible makes them attractive targets for new treatment models in both cancer and inflammatory diseases. The term epidrugs was coined by Ivanov and co-workers as drugs that inhibit or activate disease-associated epigenetic proteins ameliorating, curing, or preventing the disease [42]. In the field of cancer, there are numerous studies on the use of epidrugs, and more recently nutrients as treatment models, but at present there is a lack of research on epidrugs in relation to oral health. There is currently US Food and Drug Administration (FDA) approved epigenetic molecules being investigated in treatment of cancer [43]. However, reports are emerging on the use of epidrugs also in inflammatory diseases. In a recent review on the influence of HDAC inhibitors (HDACi) on bone remodeling [44••], it was reported that HDACi influence osteoclast differentiation, maturation and activity. In addition, it has been found that HDAC inhibitors suppress bone loss in rheumatoid arthritis (RA) as well as in periodontitis and has therefore been suggested as potential treatment models for these diseases [45, 46]. A challenge in periodontal tissue regeneration is that it involves both differentiation of cells into osteogenic cells as well as reducing inflammation. Epidrugs has been suggested as a tool for improving tissue regeneration. Treatment of periodontal ligament fibroblasts with HDACi iSodium butyrate promoted expression of osteoblast-related proteins as well as inhibited production of pro-inflammatory cytokines [47]. The use of the HDACi 1179.4b was shown to suppress alveolar bone loss, but did not suppress gingival inflammation [46]. In contrast, using the BET inhibitor JQ1 inhibits both inflammatory response and alveolar bone loss [48]. BET proteins are epigenetic regulators that interact with acetylated histones influencing the transcription machinery thereby regulating gene transcription [48].

In a recent study, it was found that periodontitis gingival tissue had an increase in mRNA expression of HDAC1, 5, 8, and 9, of these HDAC1 was found in a significantly larger amount in diseased tissue compared to non-diseased tissue [49]. Interestingly, the HDAC1 protein was found in inflammatory cells indicating a role in the regulation of inflammation [49]. Treatment of human PDL cells with HDACi Trichostatin A (TSA) resulted in a decrease in HDAC3, increase in histone H3 acetylation, and induction of osteogenic differentiation [50]. Using the DNA methylation inhibitor 5-aza-2′-deoxycytidine (5-aza) increased the responsiveness of gingival fibroblasts to TGF-β1 as well as an increase in DNMTs suggesting a new potential tool for improving wound healing and periodontal tissue regeneration [51].

### Tissue Engineering

The loss of supporting tissues in periodontitis is a major clinical challenge. To improve the prognosis of teeth with periodontitis and implants affected by peri-implantitis regeneration of the alveolar bone is needed. A goal for periodontal tissue engineering/regenerative medicine is to restore all the periodontal components, i.e., the bone, ligament, cementum, and surrounding soft tissues [52]. The two major tools used for tissue regeneration is scaffolds and gene therapy. The function of a scaffold is to provide a template, to guide the tissue and block down-growth of epithelial cells. The structure, topography, and material properties of a scaffold influence these processes. In addition, these factors also influence differentiation, migration, and adhesion of cells to the scaffold [53], factors vital to ensure a positive outcome of tissue regeneration. Recently, studies are emerging on the influence of materials as well as surface structure on epigenetic mechanisms. Cells grown on a stiff surface have a transcriptional active chromatin while cells grown on a soft material have a transcriptionally inactive chromatin [54]. Thereby, introducing epigenetics as a new treatment option in tissue engineering. It has been shown that altering a titanium dioxide surface structure at the nanoscale level altered the histone methylation pattern in human adipocytes thereby directing these cells towards osteogenic differentiation [55].

Synthetically synthesized hydroxy apatit (Hap) is considered a potential biomaterial suitable for scaffolds and implant coating. Bone marrow stromal cells and pre-osteoblasts exposed to nanosized Hap particles were shown to obtain an altered DNA methylation pattern and gene expression of ALP. Interestingly, osteoblasts in early stage of differentiation were more susceptible than later stage osteoblasts [56]. Scaffolds with nanostructured topography have been suggested as a potential tool to improve periodontal tissue engineering [57•]. Even though research on how surface topography and material energy affect the epigenome is in its beginning, the current knowledge indicates an interesting possibility to use materials and nanotechnology to promote tissue regeneration and cellular functions through epigenetics. It also shows the importance of having a knowledge of cellular functions as well as knowledge of material “structures” in order to obtain the best outcome of periodontal regeneration. Not only can the structure of a scaffold induce epigenetic changes, a scaffold can also be used as a delivery model for epidrugs. Silica is a material that has been approved by the FDA as a delivery vehicle for DNA methylation inhibitor 5-aza [58].

To improve tissue engineering, biochemical molecules are used to induce a specific function in cells or to induce differentiation of cells towards a specific cell type. By combining scaffold characteristics with delivery of epidrugs, tissue regeneration can further be improved. Human gingival fibroblasts (HGFs) present a good cell source for periodontal tissue regeneration. In a recent study by Cho and co-workers [34•], the authors induced differentiation of HGFs into osteoblasts by inducing demethylation and gene expression of osteogenic factors RUNX2 and ALP. Subsequent treatment with BMP2 resulted in fibroblast differentiation towards the osteoblast lineage and bone formation. In addition, in an in vivo mice model transplantation of these cells together with Bio-Oss bone material and Tisseel fibrin gel resulted in an increased bone mineral content and bone formation compared to controls [34•].

## Future Concepts Within the Field of Epigenetics

Epigenetics is a complex field with DNA methylation, histone acetylation and methylation linked together regulating gene expression, and just as we think we have come to understand the concept new epigenetic markers are identified. Up to now, it has been widely accepted that the 5mC/5hmC concept is the only form of DNA methylation. However, recently a new form was identified in mouse embryonic stem cells—the N^6^-methyladenin (6mA or m^6^A) [59]. This modification was associated with epigentic silencing that influence embryonic stem cell differentiation. In contrast, 6mA was previously identified in Chlamydomonas green algae but was associated with transcriptional activation since it was mostly being situated near transcription start sites. An additional function suggested for 6mA was to regulate the positioning of nucleosomes, since it was only formed on the linker DNA between nucleosomes [60].

In addition, recently a new field within epigenetics has emerged, called epitranscriptome [61•]. In addition to methylation of cytosine bases, and recently adenine bases, in the DNA it has now been discovered that adenine bases in the RNA can also become methylated [62, 63]. This epigenetic modification affects RNA stability and translation as well as RNA splicing, i.e., a mechanism that makes it possible for a cell to produce different versions of a protein from one single gene [61•]. Two variants of methylation of adenine within RNA have been identified, i.e., N^6^-methyladenosine (m6A) and the further methylated form N^6^,2-O-dimethyladenosine [64]. As for the epigenom, the epitranscriptome is dynamic and reversible and may further add to the regulation of mRNA transcription and gene expression.

## Conclusion

Several key factors, such as diet, smoking, bacteria, and inflammation, known to induce epigenetic alterations, come in contact with the oral mucosa and may affect the oral health. Even though research has emerged on the influence of bacteria and inflammation, knowledge of how diet and smoking influence epigenetic mechanisms in the oral mucosa is lacking. Periodontitis is a complex disease with a mosaic of cells, cytokines, and signaling pathways involved in the activation and regulation of the immune response and tissue destruction. Knowledge of epigenetic pattern in periodontal diseases may add not only to the knowledge of susceptibility of the disease but may also be a diagnostic tool to identify patients at risk to develop the severe form of periodontitis. In addition, recent research within gene therapy and tissue engineering indicate a role for epigenetics also to improve regeneration of periodontal tissues.

## References

[1] Kornman KS (2008). Mapping the pathogenesis of periodontitis: a new look. J Periodontol.

[2] Hugosson A, Sjödin B, Norderyd O (2008). Trends over 30 years, 1973-2003, in the prevalence and severity of periodontal diseases. J Clin Periodontol.

[3] Eke PI, Dye BA, Wei L, Thornton-Evans GO, Genco RJ (2012). Prevalence of periodontitis in adults in the United States: 2009 and 2010. J Dent Res.

[4] Kassebaum NJ, Bernabé E, Dahiya M, Bhandari B, Murray CJ, Marcenes W (2014). Global burden of severe periodontitis in 1990-2010: a systematic review and meta-regression. J Dent Res.

[5] Meyle J, Chapple I (2015). Molecular aspects of the pathogenesis of periodontitis. Periodontol.

[6] Offenbacher S, Barros SP, Beck JD (2008). Rethinking periodontal inflammation. J Periodontol.

[7] Larsson L, Castilho RM, Giannobile WV (2015). Epigenetics and its role in periodontal diseases—a state-of-the-art review. J Periodontol.

[8] Lod S, Johansson T, Abrahamsson KH, Larsson L (2014). The influence of epigenetics in relation to oral health. Int J Dent Hygiene.

[9] Seo J-Y, Park Y-J, Yi Y-A, Hwang J-Y, Lee I-B, Cho B-H (2015). Epigenetics: general characteristics and implications for oral health. Restor Dent Endod.

[10] Waddington CH (2012). The epigenotype. Endeavor 1942:1:18-20. Reprinted in Int J Epidemiol.

[11] Bird A (2002). DNA methylation patterns and epigenetic memory. Genes Dev.

[12] Jenuwein T, Allis CD (2001). Translating the histone code. Science.

[13] Robertson KD, Wolffe AP (2000). DNA methylation in health and disease. Nat Rev Genet.

[14] Tahiliani M, Koh KP, Shen UY, Pastor WA, Bandukwala H, Brudno Y (2009). Conversion of 5-methylcytosine to 5-hydroxymethylcytosine in mammalian DNA by MLL partner TET1. Science.

[15] Kraus TFJ, Globisch D, Wagner M, Eigenbrod S, Widmann D, Münzel M (2012). Low values of 5-hydroxymethylcytosine (5hmC), the sixth base, are associated with anaplasia in human brain tumors. Int J Cancer.

[16] Barros SP, Offenbacher S (2014). Modifiable risk factors in periodontal disease. Epigenetic regulation of gene expression in the inflammatory response. Periodontol.

[17] Martins MD, Jia Y, Larsson L, Almeida LO, Garaicoa-Pazmino C, Le JM (2016). Epigenetic modifications of histones in periodontal disease. J Dent Res.

[18] Benakanahere M, Abdolhosseini M, Hosur K, Finoti LS, Kinane DF (2015). TLR2 promoter hypermethylation creates innate immune dysbiosis. J Dent Res.

[19] De Oliveira NF, Andia DC, Planello AC, Pasetto S, Marques MR, Nociti JRFH (2011). TLR2 and TLR4 gene promoter methylation status during chronic periodontitis. J Clin Periodontol.

[20] de Faria Amormino SA, Arao TC, Saraiva AM, Gomez RS, Dutra WO, da Costa JE (2013). Hypermethylation and low transcription of TLR2 gene in chronic periodontitis. Human Immunobiol.

[21] Abu-Amer Y (2013). NF-kappaB signaling and bone resorption. Osteoporosis Int.

[22] Takai R, Uehara O, Harada F, Utsunomiya M, Chuju T, Yoshida K (2016). DNA hypermethylation of extracellular matrix-related genes in human periodontal fibroblasts induced by stimulation for a prolonged period with lipopolysaccharide derived from *Porhyromonas gingivalis*. J Periodontal Res.

[23] Miao D, Godovikova V, Qian X, Seshadrinathan S, Kapila YL, Fenno JC (2014). *Treponema denticola* upregulates MMP-2 activation in periodontal ligament cells: interplay between epigenetics and periodontal infection. Arch Oral Biol.

[24] Franco C, Hernandez-Rios P, Timo S, Biguetti C, Hernandez M (2017). Matrix metalloproteinases as regulators of periodontal inflammation. Int J Mol Sci.

[25] De Souza AP, Planello AC, Marques MR, De Carvalho DD, Line SRP (2014). High-throughput DNA analysis shows the importance of methylation in the control of immune inflammatory gene transcription in chronic periodontitis. Clin Epigenet.

[26] Schulz S, Immel UD, Just L, Schaller H-G, Gläser C, Reichert S (2016). Epigenetic characteristics in inflammatory candidate genes in aggressive periodontitis. Human Immunol.

[27] Stefani FA, Viana MB, Dupim AC, Brito JAR, Gomez RS, da Costa JE (2013). Expression, polymorphism and methylation pattern of interleukin-6 in periodontal tissues. Immunobiology.

[28] Kobayashi T, Ishida K, Yoshie H (2016). Increased expression of interleukin-6 (IL-6) gene transcript in relation to IL-6 promoter hypomethylation in gingival tissue from patients with chronic periodontitis. Arch Oral Biol.

[29] Kojima A, Kobayashi T, Ito S, Murasawa A, Nakazono K (2016). Tumor necrosis factor-alpha gene promoter methylation in Japanese adults with chronic periodontitis and rheumatoid arthritis. J Periodontal Res.

[30] Baptista NB, Portinho D, Casarin RCV, Vale HF, Casati MZ, De Souza AP (2014). DNA methylation levels of SOCS1 and LINE-1 in oral epithelial cells from aggressive periodontitis patients. Arc Oral Biol.

[31] Andia DC, Planello AC, Portinho D, Sa Silva RA, Salmon CR, Sallum EA (2015). DNA methylation analysis of SOCS1, SOCS3 and LINE-1 in microdissected gingival tissue. Clin oral invest.

[32] Planello AC, Singhania R, Kron KJ, Bailey SD, Ronlois D, Lupien M (2016). Pre-neoplastic epigenetic disruption of transcriptional enhancers in chronic inflammation. Oncotarget.

[33] Uehara O, Abiko Y, Saitoh M, Miyakawa H, Nakazawa F (2014). Lipopolysaccharide extracted from Porphyromonas gingivalis induces DNA hypermethylation of runt-related transcription factor 2 in human periodontal fibroblasts. J Microbiol, immunol Infect.

[34] • Cho Y, Kim B, Bae H, Kim W, Baek J, Woo K, et al. Direct gingival fibroblast/osteoblast transdifferentiation via epigenetics. J Dent Res. 2017; 10.1177/0022034516686745. **This study demonstrates the possibility of differentiate human gingival fibroblast into functional osteoblasts using epigenetics.**

[35] Larsson L, Thorbert-Mros S, Lopez-Lago A, Kalm J, Shikhan A, Berglungh T (2016). Expression of TET2 enzyme indicates enhanced epigenetic modification of cells in periodontitis. Eur J Oral Sci.

[36] Jiang R, Jones MJ, Chen E, Neumann SM, Fraser HB, Miller GE (2015). Discordance of DNA methylation variance between two accessible human tissues. Sci Rep.

[37] Sadakierska-Chudy A, Kostrzewa RM, Filip M (2015). A comprehensive view of the epigenetic landscape part I: DNA methylation, passive and active DNA demethylation pathways and histone variants. Neurotox Res.

[38] Kusumoto T, Hamada T, Yamada N, Nagata S, Kanmura Y, Houjou I (2012). Comprehensive epigenetic analysis using oral rinse samples: a pilot study. J Oral Maxillofac Surg.

[39] Nagata S, Hamada T, Yamada N, Yokoyama S, Kitamoto S, Kanmura Y (2012). Aberrant DNA methylation of tumor-related genes in oral rinse. Cancer.

[40] Eipel M, Mayer F, Arent T, Ferreira MRP, Birkhofer C, Gerstenmaier U (2016). Epigenetic age predictions based on buccal swabs are more precise in combination with cell type-specific DNA methylation signatures. Aging.

[41] Langie SAS, Moisse M, Declerck K, Koppen G, Godderis L, Vanden Berghe W, et al. Salivary DNA methylation profiling: aspects to consider for biomarker identification. Basic Clin Pharmacol Toxicol. 2016; 10.1111/bcpt.12721. 10.1111/bcpt.12721 PMC5644718 27901320

[42] Ivanov M, Barragan I, Ingelman-Sundberg M (2014). Epigenetic mechanisms of importance for drug treatment. Trends Pharmacol Sci.

[43] Martins MD, Castilho RM (2013). Histones: controlling tumor signaling circuity. J carcinog Mutagen.

[44] Cantley MD, Zannettino ACW, Bartold PM, Fairlie DP, Haynes DR (2017). Histone deacetylases (HDAC) in physiological and pathological bone remodelling. Bone.

[45] Cantley MD, Fairlie DP, Bartold PM, Rainsford KD, Le GT, Lucke AJ (2011). Inhibitors of histone deacetylases in class I and class II suppress human osteoclasts in vitro. J Cell Physiol.

[46] Cantley MD, Bartold PM, Marino V, Fairlie LGT, Lucke AJ (2011). Histone deacetylase inhibitors and periodontal bone loss. J Periodontal Res.

[47] Kim T-I, Han J-E, Jung H-M, Oh J-W, Woo KM (2013). Analysis of histone deacetylase inhibitor-induced responses in human periodontal ligament fibroblasts. Biotechnol Lett.

[48] Meng S, Zhang L, Tang Y, Tu Q, Zhen L, Yu D (2014). BET inhibitor JQ1blocks inflammation and bone destruction. J Dent Res.

[49] Cantley MD, Dharmapatni AA, Algate K, Crotti TN, Bartold PM, Haynes DR (2016). Class I and II histone deacetylase expression in human chronic periodontitis gingival tissue. J periodont Res.

[50] Huynh NC-N, Everts V, Pavasant P, Ampornaramveth RS (2016). Inhibition of histone deacetylases enhances the osteogenic differentiation of human periodontal ligament cells. J Cell Biochem.

[51] Sufarn I-G, Beikircher G, Weinhausel A, Gruber R (2017). Inhibitors of DNA methylation support TGF-b1-induced IL11 expression in gingival fibroblasts. J Periodontal Implant Sci.

[52] Larsson L, Decker AM, Nibali L, Pilipchuk SP, Berglundh T, Giannobile WV (2016). Regenerative medicine for periodontal and peri-implant diseases. J Dent Res.

[53] Nair A, Tang L. Influence of scaffold design on host immune and stem cell responses. Semin Immunol. 2017; 10.1016/j.smim.2017.03.001. 10.1016/j.smim.2017.03.001 28431919

[54] Rabineau M, Flick F, Mathieu E, Tu A, Senger B, Voegel JC (2015). Cell guidance into quiescent state through chromatin remodeling induced by elastic modulus of substrate. Biomaterials.

[55] Lv L, Liu Y, Zhang P, Zhang X, Liu J, Chen T (2015). The nanoscale geometry of TiO2 nanotubes influences the osteogenic differentiation of human adipose-derived stem cells by modulating H3K4 trimethylation. Biomaterials.

[56] Ha S-W, Jang HL, Nam KT, Beck JRGR (2015). Nano-hydroxyapatite modulates osteoblast lineage commitment by stimulation of DNA methylation and regulation of gene expression. Biomaterials.

[57] Du M, Duan X, Yang P (2015). Induced pluripotent stem cells and periodontal regeneration. Curr Oral Health Rep.

[58] Lorden ER, Levinson HM, Leong KW (2001). Integration of drug, protein, and gene delivery systems with regenerative medicine. Drug Deliv Transl Res.

[59] Wu TP, Wang T, Seetin MG, Lai Y, Zhu S, Lin K (2016). DNA methylation on N6-adenine in mammalian embryonic stem cells. Nature.

[60] Fu Y, Luo G-Z, Chen K, Deng X, Yu M, Han D (2015). N^6^-Methyldeoxyadenosine marks active transcription start sites in *Chlamydomonas*. Cell.

[61] Willyard C (2017). A new twist on epigenetics. Nature.

[62] Meyer KD, Saletore Y, Zubo P, Elemento O, Mason CE, Jaffrey SR (2012). Comprehensive analysis of mRNA-methylation reveals enrichments in 2′ UTRs and near stop codons. Cell.

[63] Dominissini D, Moshitch-Moshkovitz S, Schwartz S, Salmon-Divon M, Ungar L, Osenberg S (2012). Topology of the human and mouse m^6^A RNA methylomes revealed by m^6^A-seq. Nature.

[64] Mauer J, Luo X, Blanjoie A, Jiao X, Grozhik AV, Patil DP (2017). Reversible methylation of m^6^Am in the 5′ cap controls mRNA stability. Nature.

